# Approaching the challenges of MKK3/p38delta MAPK targeting for therapeutic purpose in colorectal cancer

**DOI:** 10.1186/s13046-019-1513-4

**Published:** 2019-12-27

**Authors:** Lorenzo Stramucci, Gianluca Bossi

**Affiliations:** Oncogenomic and Epigenetic Unit, Department of Diagnostic Research and Technological Innovation, IRCCS - Regina Elena National Cancer Institute, Via Elio Chianesi 53, 00144 Rome, Italy

**Keywords:** Colorectal Cancer, MKK3 / p38 MAPK signalling, p38 delta MAPK, Chemotherapy, Target therapy, Combined treatments

## Abstract

MKK3 is a member of the dual specificity kinase group specific upstream activator of p38 MAPK proteins. We originally identified MKK3 as mutant p53 (mutp53) gain-of-function (GOF) upregulated target gene in different tumor models. To deeply investigate the MKK3 functions in cancer, taking advantage of a panel of authenticated colorectal cancer (CRC) lines and primary colonocytes, we found that MKK3 activates specifically p38delta MAPK protein, which signaling is further triggered by 5-fluorouracil (5-FU) treatments, a largely adopted chemotherapeutic drug in CRC clinical practice. The overall achieved results proposed the MKK3/p38delta MAPK as relevant molecular axis involved in abrogating efficacy to 5-FU treatments in CRC. This commentary will provide an overall discussion of the results that have been achieved contextualizing them in the overview of the knowledge in the p38 MAPK field in cancer disease.

## Background

Colorectal cancer (CRC) is one of the most common malignant tumor worldwide, thus the understanding of its underlying molecular mechanisms is crucial for the development of therapeutic strategies. The Mitogen Activated Protein Kinase-Kinase 3 (MKK3) belongs to a dual specificity kinase group (MKK) and is activated by a wide array of upstream kinases (MEKK1–4) through Ser-189 and Thr-193 phosphorylation sites. MKK3 serves, together with MKK6, as a specific activator of p38 Mitogen Activated Protein Kinase (MAPK) family members (alpha, beta, delta and gamma) [1], through which contributes to the regulation of several cellular functions such as proliferation, differentiation, apoptosis as well as response to drugs. At present, the exact MKK3/p38 MAPK pathway contribution in cancer is heavily debated because of its pleiotropic functions. In a recently published work [2], our group uncovered novel molecular mechanisms through which MKK3 supports proliferation and survival in CRC, further supporting MKK3 as a novel and extremely attractive therapeutic target for the development of promising strategies for the management of CRC patients.

## Main text

We originally identified and validated MKK3 as a mutant p53 transcriptional target gene involved in the acquisition of novel oncogenic functions (GOF, gain-of-funciton) through which mutant p53 actively sustains tumor malignancy [3]. We thereafter demonstrated that MKK3 exerted relevant pro-survival functions even in p53 wild-type cellular contexts and that its epigenetic inactivation results into anti-proliferative and pro-apoptotic effects in tumor cells but not in normal cells [4], suggesting that MKK3 targeting could represent a therapeutically intriguing strategy [5]. However, when it comes to translating MKK3 targeting for clinical use, the controversial role exerted by p38 MAPK proteins, the MKK3 immediate downstream mediators [1], poses practical issues and hence ethical caveats. Indeed, p38 MAPK phosphorylation and activation is observed in response to a variety of stimuli resulting into contradictive effects [1]. Furthermore, evidence suggests that the pleiotropic effects reported for the p38 MAPK pathway do not simply rely on phosphorylation by upstream kinases and of downstream substrates, but rather isotype-specific p38 MAPK activation, auto-phosphorylation events, protein-protein interactions, as well as the cellular and molecular context in which p38 MAPK activation occurs, all contribute to skew the final outcome of p38 MAPK signaling activation [1]. Hence, a deeper characterization of the diverse players involved and their fine network of interactions with the p38 MAPK node is indispensable to define and correctly predict the outcome of the p38 MAPK pathway manipulation.

**Fig. 1:**
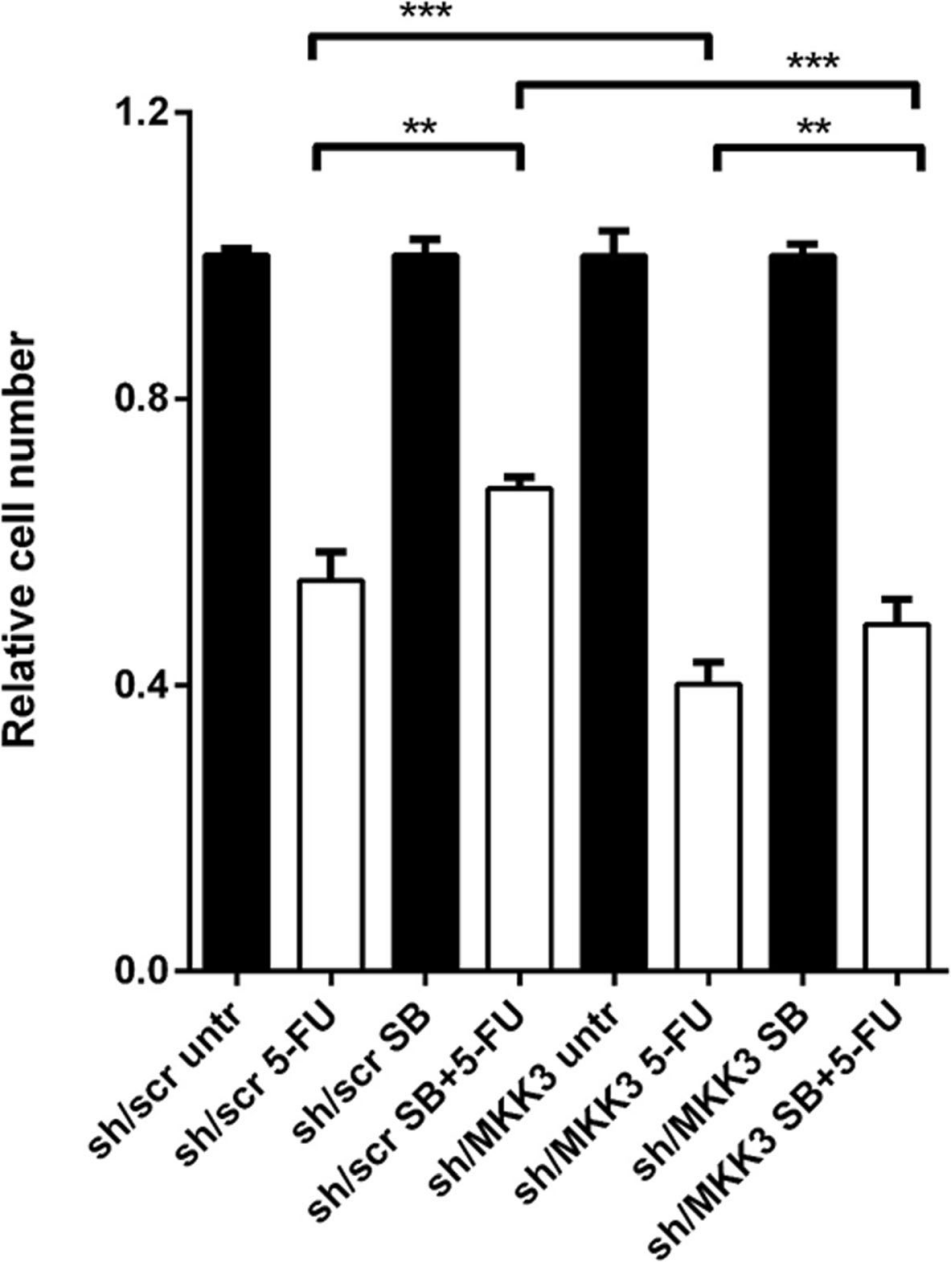
The p38alpha MAPK pharmacological inhibition by SB203580 treatment hinders response to 5-FU. The HT29-sh/scr and -sh/MKK3 sublines were pre-treated (72 h) with doxycycline to induce sh/RNA expression, then exposed to either 5-FU (20 μM, 6 h), SB203580 (10 μM, 24 h) or their combination. Live cells were quantified by crystal violet staining 72 h after exposure. Viability was normalized to their respective sh/scr control set to 1.0. Means and Standard Deviations of results from two independent experiments are reported. Significance was assessed by two-tailed paired t test using Graph Pad Prism Software. **p < 0.01, ***p < 0.001

In this view, taking advantage of a panel of colorectal cancer (CRC) lines, we found [2] that interestingly, MKK3 activates specifically p38delta MAPK isotype, and that this molecular signaling is triggered further by challenging CRC cells with 5-Fluorouracil (5-FU) hampering its efficacy. Noteworthy, 5-FU is currently the cornerstone of treatment for CRC patients [6]. According with these evidences, the anti-tumor effects observed upon MKK3 depletion in CRC lines are largely exerted through the selective inhibition of the p38delta MAPK isotype. In fact, p38delta MAPK ablation mimicked MKK3 knockdown effects and impaired CRC cell growth and boosted 5-FU efficacy in vitro and in vivo, but did not negatively affect healthy colonocytes [2], indicating that the MKK3/p38delta MAPK axis inhibition could represent an excellent target in CRC management. By contrast, although not univocally, the p38alpha MAPK isotype has been reported to exert anti-tumoral effects in CRC [7], and, in agreement with that, we were able to confirm that p38alpha MAPK pharmacological inhibition (SB203580) hinders response to 5-FU (Fig. 1). Strikingly, the protective effect of SB203580 was still observed in MKK3 depleted CRC cells (Fig. 1), underlining that tumor suppressive p38alpha MAPK signaling persists when the tumor supportive MKK3/p38delta MAPK signaling is inhibited, and that, at least in vitro the net biological outcome of MKK3 inhibition is mainly driven by the blockade of pro-survival signals.

Such observations, performed in the same CRC models, highlights that the ideal manipulation of the p38 MAPK pathway for therapeutic purposes should aim at selective inhibition of those p38 MAPK signaling arms responsible for pro-tumoral effects while leaving those responsible for anti-tumor effects unaffected. Specifically, in CRC while p38delta MAPK targeting is desired, p38alpha MAPK should remain active. However, when it comes to already available therapeutic tools, it is to be acknowledged that p38 MAPK inhibitors have been developed to target specifically the p38alpha MAPK isoform because of its wide tissue distribution and abundance. As a result, currently available p38 MAPK targeting drugs only display partial isotype selectivity [1] making the pharmacologic inactivation of a particular p38 MAPK isotype a moving target to be hit. Indeed, in the precise CRC scenario, BIRB-796 treatment, whose antitumor efficacy is currently being explored in clinical trials, could theoretically target the p38delta MAPK (for which an IC_50_ > 100 nM is reported), but that would simultaneously target the p38alpha MAPK (IC_50_ = 4 nM) [8], resulting into unpredictable and probably unwanted outcomes. In this perspective, targeting the immediately upstream p38 MAPK kinase, the MKK3 that, at least to some extent, such as in our identified MKK3/p38delta MAPK prosurvival signaling axis in CRC, display a discrete degree of substrate specificity [9] could also be an option. Indeed, promising MKK3 inhibitors have recently been developed [10] and the evaluation of their potential in preclinical settings will likely provide evidence of their suitability for a more tailored inhibition of the p38 MAPK pathway.

## Conclusions

Overall, the identified molecular mechanisms, involving MKK3 in supporting proliferation and survival signaling in CRC, suggest MKK3 as a novel and extremely attractive therapeutic target for the development of promising strategies for the management of CRC patients.

## Data Availability

Not applicable.

## Abbreviations

5-FU: 5-Fluorouracil
CRC: Colorectal cancer
GOF: Gain-of-function; IC50: inhibitory concentration
MKK3: Mitogen Activated Protein Kinase-Kinase 3
p38 MAPK: Mitogen Activated Protein Kinase
